# Gut Microbiota as an Innovative Therapeutic Target in Cardiovascular Diseases from a Metabolic and Inflammatory Perspective

**DOI:** 10.3390/biomedicines14061267

**Published:** 2026-06-01

**Authors:** Emília Hijová, Izabela Bertková, Veronika Benetinová

**Affiliations:** Center of Clinical and Preclinical Research, MediPark, Faculty of Medicine, Pavol Jozef Šafárik University in Košice, SNP 1, 040 11 Košice, Slovakia; izabela.bertkova@upjs.sk (I.B.); veronika.benetinova@upjs.sk (V.B.)

**Keywords:** gut microbiota, cardiovascular diseases, microbial metabolites

## Abstract

The gut microbiome plays a key role in the pathogenesis of cardiovascular disease through systemic inflammation, impaired lipid metabolism, and proatherogenic gut metabolites like trimethylamine N-oxide. Gut dysbiosis contributes to decreased level of microbial metabolites such as short-chain fatty acids, bile acids, coprostanol, and phenylacetylglutamine, as well as increased intestinal permeability and platelet hyper-reactivity, and exacerbating cardiovascular risk. New microbiome-focused treatments such as probiotics, prebiotics, synbiotics, and fecal microbiota transplantation are showing potential to help reduce cardiovascular diseases. However, bringing these therapies into clinical settings is difficult because they vary by strain and individual response. The gut–heart connection offers an innovative approach to preventing and treating heart condition, but additional research is needed to ensure lasting effectiveness and safety.

## 1. Introduction

Cardiovascular diseases (CVDs), which comprise a range of disorders affecting the heart and blood vessels, are the primary cause of mortality and disability within the European region. Recent data indicates that CVD resulted in 2.2 million deaths among women and 1.9 million deaths among men [1]. The *European Society of Cardiology’s Atlas of Cardiology* report analyzed CVD data across 57 member countries. Cardiovascular diseases are responsible for 47% deaths among women and 39% among men in these regions. Ischemic heart disease causes 38% of CVD-related deaths in men and 47% in women, while stroke is the second leading cause—accounting for 26% of CVD deaths in women and 21% in men. In Slovakia, CVD remains the foremost cause of mortality, comprising 45.3% of all deaths in 2022, with 40.7% in men and 50.3% in women [2].

Cardiovascular diseases present significant clinical challenges, with their pathogenesis influenced by a range of factors. Emerging evidence indicates that gut microbiota and their metabolites play crucial roles in maintaining homeostasis and are implicated in disease susceptibility through modulation of host metabolic and immune functions. The composition of gut microbiota is determined by genetic background, as well as environmental influences such as diet and lifestyle, exerting a substantial impact on both the immune and metabolic systems of the host. This underscores the close relationship between gut microbiota, human health, and the development of various diseases [3,4]. Consequently, the gut microbiota constitutes a promising target for intervention via nutritional strategies and supplementation with natural compounds, increasingly recognized as an integral aspect of health management and as a potential therapeutic target for numerous conditions, including CVD [5,6,7].

Gut dysbiosis, or changes in gut microbial structure and function, is linked to various diseases, including cardiovascular disorders [8,9]. An imbalance—often due to diet and obesity—increases CVD risk through altered levels of metabolites like short-chain fatty acids (SCFAs) [10], trimethylamine N-oxide (TMAO) [11], bile acids (BAs) [12], coprostanol [13], and phenylacetylglutamine (PAGln) [14].

Clinical research in the framework of controlling CVD from the gut microbiota changes must be focused on the development of innovative and cost-effective methods for the prevention and treatment of CVD. Microbiome-targeted therapies (including probiotics, prebiotics, and synbiotics), fecal microbiota transplantation (FMT), and dietary interventions, show promise in alleviating CVD.

This review article first provides an overview of the current state of research on the relationship between CVD and gut microbiota with respect to various risk factors, summarizes the role of bacterial metabolites and the immune system, and, finally, discusses possible future directions and intervention in this area of the research to lower cardiovascular risk.

## 2. Risk Factors for Cardiovascular Diseases

Cardiovascular diseases account for approximately 17 million deaths each year, constituting about 31% of all global fatalities. The World Health Organization has projected this figure to rise beyond 23 million by 2030 [15]. Conditions such as CVD, including coronary artery disease, atherosclerosis, hypertension, and heart failure, remain significant contributors to mortality and represent substantial economic and health challenges worldwide.

The main risk factors for CVD can be divided into the following:

A. Environmental risk factors—air pollution (exposure to heavy metals, pesticides, polycyclic aromatic hydrocarbons) and noise contribute to more than 75% of the CVD, can significantly alter the gut microbiota, induce gut dysbiosis, promote inflammation, and disrupt metabolic processes [16,17].
B. Health behavior—smoking, lower physical activity/a sedentary lifestyle, dietary factors (high-fat diet rich in choline and L-carnitine, increased consumption of salt and trans fatty acids, sugar, sugar-sweetened beverages, and low amounts of vegetables, fruits and fibers) [18,19] all contribute.
C. Clinical risk factors—elevated blood pressure (≥140/90 mmHg); increased prevalence of obesity with body mass index (BMI ≥ 30 kg/m^2^) associated with diabetes mellitus, recognized as one of the major global health challenges of the 21st century; and a high level of total cholesterol, as well as a non-high-density lipoprotein cholesterol level higher than 2.6 mmol/L (or, in case of diabetes mellitus, higher than 2.2 mmol/L) are key contributors [1].

Cardiovascular diseases, as non-communicable conditions, share modifiable risk factors such as diet and physical activity. Integrating their prevention into population-wide strategies can reduce disability, death, and improve quality of life.

## 3. Gut–Heart Axis

The gut–heart axis represents a complex, multi-mechanistic communication system that extends beyond the direct effects of the gut microbiota and its metabolites. It involves interconnected pathways such as microbial metabolites, immune signaling, neural communication, endocrine mechanisms, and intestinal barrier integrity. The immune system and inflammation-related signaling also play a significant role in this context, with chronic low-grade inflammation representing one of the key mechanisms linking gut dysbiosis with cardiovascular diseases.

By better understanding the interaction between the gut microbiome and the cardiovascular system, we can come up with therapeutic approaches and general recommendations that would help exert the cardioprotective role of the gut microbiome. The human gut microbiota is considered a distinct organ, with significant interactions within the host’s biological functions, and plays an active role in various metabolic, immunological and endocrine responses [20]. The gut microbiota is represented by the bacterial phyla *Firmicutes*, *Bacteroidetes*, *Actinobacteria*, *Proteobacteria*, *Fusobacteria*, and *Verrucomicrobia*, with *Firmicutes* and *Bacteroidetes* being the predominant types [21] which play an important role in maintaining the health of the body. The composition and function of the gut microbiota is dynamic and affected by diet properties. The gut microflora can form the intestinal epithelial barrier and regulate immune functions, vitamin production, and nutrient digestion, and prevent pathogen invasion [22,23].

The gut microbiome is linked to CVD in the following ways:

- ***directly, through the production of metabolites, and***
- ***indirectly, affecting the immune system through immunomodulation***

**Figure 1:**
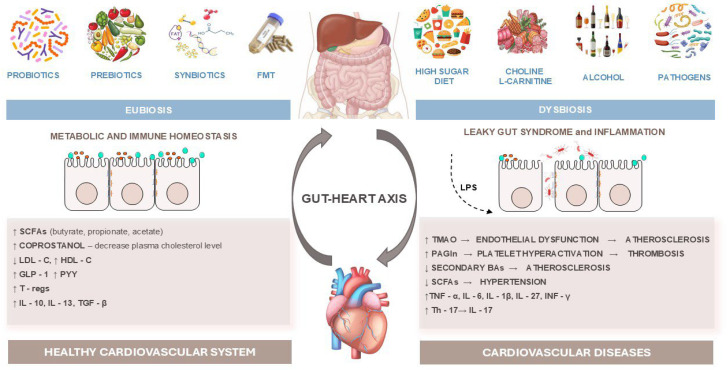
The Gut–Heart Axis: eubiosis and dysbiosis in intestinal homeostasis and cardiovascular health.

Changes in the gut microbiome, resulting from either an excess of harmful, or lack of beneficial, metabolites, may have an impact on the cardiovascular system. Gut dysbiosis is characterized by a deviation from the healthy state, termed eubiosis (Figure 1). Dysbiosis in the gut microbiome is manifested by changes in the composition and function of the microbiome, reduced microbial diversity, and an imbalance between beneficial and pathogenic microbiota [24,25]. Altered microbial composition impacts systemic health by promoting metabolic and inflammatory disorders, especially cardiovascular disease [26,27,28]. Dysbiosis can trigger systemic inflammation through various mechanisms, such as lipopolysaccharide (LPS) production [29], activation of nucleotide-binding oligomerization binding domain-1 (NOD1), nucleotide-binding oligomerization binding domain-2 (NOD2) and NOD-like receptor protein-3 (NLRP3) inflammasome [30], increased leukocyte count [31], and increased serum level of various inflammatory cytokines [32].

The gut–heart axis refers to the bidirectional relationship between intestinal microbiota and the cardiovascular system, mediated by microbial metabolites, immune signaling, and gut barrier integrity. Eubiosis, achieved through interventions such as probiotics, prebiotics, synbiotics, and FMT, enhances metabolic and immune homeostasis via SCFAs, coprostanol, GLP-1, PYY, anti-inflammatory cytokines, and Tregs, leading to strengthened epithelial barriers, ↓ systemic inflammation, ↓ LDL-C, ↑ HDL-C, and overall cardiovascular protection. Dysbiosis, due to high-sugar diets, choline-/L-carnitine-rich foods, alcohol consumption, or pathogenic overgrowth, impairs gut barrier integrity and promotes a pro-inflammatory state. ↑ Gut permeability allows LPS translocation, which triggers chronic low-grade inflammation, endothelial dysfunction, platelet hyperreactivity, thrombosis, hypertension, atherosclerosis, and ↑ microbial TMAO and PAGln. ↓ SCFAs and secondary BAs further exacerbate cardiovascular risk, indicating the direct influence of gut microbiota on cardiovascular disease pathogenesis.

Abbreviations: BAs, bile acids; FMT, fecal microbiota transplantation; GLP-1, glucagon-like peptide-1; HDL-C, high-density lipoprotein cholesterol; IFN, interferon; IL, interleukin; LDL-C, low-density lipoprotein cholesterol; LPS, lipopolysaccharide; PAGln, phenylacetylglutamine; PYY, peptide YY; SCFAs, short-chain fatty acids; TMAO, trimethylamine N-oxide; TNF, tumor necrosis factor; Tregs, regulatory T cells; ↑: increase; ↓: decrease, →: leads to.

### 3.1. Mechanisms Linking Gut Microbiota to Cardiovascular Diseases—Direct Impact of Microbial Metabolites

The gut microbiota plays a key role in host physiology, regulating numerous metabolic processes including the lipid and glucose metabolism, digestion and fermentation of non-digestible substrates, and vitamin synthesis [33,34]. These processes lead to the production or modulation of metabolites by the microbiota that act as metabolic substrates and signaling molecules, which has important implications for host health [35]. Microbial metabolites have different functions, some of which may be harmful and negatively impact cardiovascular health such as TMAO, while others, such as SCFAs, coprostanol, phenylacetylglutamine, or bile acids, may be beneficial and have a protective role.

a. **Trimethylamine N-oxide**

**Figure 2:**
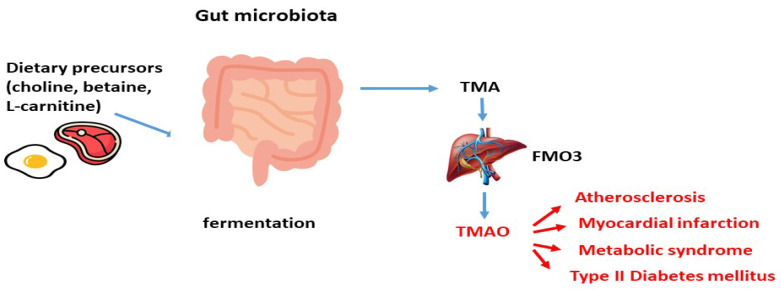
TMAO production and its effect on cardiovascular and metabolic diseases. More details are included in the main text.

Trimethylamine N-oxide (TMAO), a proatherogenic gut microbe-dependent metabolite, is associated with a higher risk of CVD and cardiorenal disorders, including atherosclerosis, hypertension, ischemic stroke, heart failure, acute myocardial infarction, chronic kidney disease, and some metabolic disorders [36]. Trimethylamine N-oxide is formed from dietary precursors such as choline, phosphatidylcholine, betaine, and L-carnitine, which are abundant in red meat, eggs, and dairy products. The gut microbiota converts these precursors into trimethylamine (TMA) via TMA lyase enzymes, including choline TMA lyase encoded by the *cutC/D* genes, carnitine TMA lyase encoded by the *cntA/B* genes, and betaine reductase encoded by the *grdH* gene) [37,38]. The ability to produce TMA has been described in various species, such as *Anaerococcus hydrogenalis*, *Clostridium asparagiforme*, *Clostridium hathewayi*, *Clostridium sporogenes*, *Edwardsiella tarda*, *Escherichia fergusonii*, *Proteus penneri*, and *Providencia rettgeri* [39,40]. Trimethylamine is absorbed into host portal circulation and oxidized by hepatic enzyme flavin monooxygenases, particularly flavin monooxygenase 3 (FMO3), producing trimethylamine N-oxide (TMAO) in the liver, TMAO is excreted by the kidneys [41,42]. Trimethylamine N-oxide has been shown to be a significant contributor to CVD (Figure 2).

Elevated TMAO levels are associated with endothelial dysfunction, modulation of cholesterol and BAs metabolism by inhibiting reverse cholesterol transport and BAs synthesis and composition. These factors promote lipid accumulation in macrophages and vascular inflammation, and foam cell formation, which contributes to atherogenesis and the development of atherosclerosis [43,44]. TMAO promotes vascular inflammation and endothelial cell injury by activating the NOD-like receptor protein 3 (NLRP3) inflammasome [45]. TMAO has been shown to stimulate the nuclear transcription factor kappa B (NF-κB) signaling pathway, partly through pathways involving high-mobility group box 1 (HMGB1), which acts as an important inflammatory mediator and contributes to increased expression of proinflammatory genes responsible for producing adhesion molecules, chemokines, and cytokines [46,47].

It has been shown that elevated TMAO levels increase platelet sensitivity to agonists, thereby increasing the risk of thrombosis, which contributes to cardiovascular complications [44] and higher rates of serious adverse cardiovascular events, including myocardial infarction and stroke [48,49].

Obesity is a leading contributor to cardiometabolic risk, including insulin resistance, atherosclerosis, and inflammation. A study by Pescari [50] reported a positive association between circulating TMAO levels and increased body weight, abdominal fat, insulin resistance, and hepatic steatosis, in 60 adult subjects. Furthermore, its correlation with carotid intima–media thickness, an early marker of arterial wall changes, supports its potential contribution to atherosclerotic progression. Western diet-induced obesity contributed to increased circulating TMAO levels, cardiac dysfunction, increased expression of the pro-inflammatory cytokine’s tumor necrosis factor-alpha (TNF-α) and interleukin (IL) IL-1β, decreased expression of the anti-inflammatory cytokine IL-10, and increased interstitial fibrosis in the heart. Administration of the TMA formation inhibitor 3,3-dimethyl-1-butanol was shown to prevent the reduction in circulating TMAO levels and to attenuate cardiac inflammation and fibrosis [51]. Alterations in gut microbiota composition, together with excessive formation of microbiota metabolites, are considered important contributors to the development of hypertension. Certain bacterial genera, including *Bifidobacterium*, *Lactobacillus*, *Streptococcus* and *Escherichia coli*, can produce neurotransmitters within the autonomic nervous system, thereby influencing vascular tone and contributing to hypertension [52]. Elevated TMAO levels have been positively associated with an increased risk of hypertension [53]. Moreover, TMAO has been shown to exert deleterious effects on adult cardiomyocytes by disrupting the T-tubule network and impairing calcium handling [54]. TMAO may contribute to myocardial dysfunction through activation of pro-inflammatory signaling pathways, enhancement of oxidative stress, promotion of myocardial fibrosis, endothelial dysfunction, and adverse ventricular remodeling. Experimental and clinical evidence further suggests its involvement in impaired myocardial energy metabolism, cardiomyocyte hypertrophy, and dysregulation of calcium homeostasis, which may ultimately lead to progressive left ventricular dysfunction and poorer clinical outcomes in patients with heart failure [55].

However, circulating TMAO levels are determined not only by cardiometabolic status, but also by the availability and bioaccessibility of dietary precursors and their modulation by food processing and gut microbial activity. Dietary precursors of trimethylamine N-oxide (TMAO), particularly choline and L-carnitine, are significantly influenced by food processing technologies, which alter their chemical structure, matrix binding, and intestinal bioavailability, thereby modulating microbial trimethylamine (TMA) production and systemic TMAO levels [56,57]. Thermal processing methods such as roasting, grilling, and frying enhance the release of phospholipid-bound choline and carnitine through disruption of cellular structures and protein denaturation, potentially increasing microbial substrate availability compared with boiling, where water-soluble compounds are partially lost by leaching [58,59]. High-temperature cooking additionally promotes lipid oxidation and advanced glycation end-product formation, which may indirectly affect gut microbial composition and cardiometabolic homeostasis [60]. Fermentation and pickling further modify precursor availability through microbial enzymatic activity and acidification, with some fermented foods reducing the abundance of TMA-producing bacteria, depending on microbial strains [61]. Overall, current evidence highlights food processing as a key determinant of TMAO metabolism, which may partly explain inconsistencies between dietary intake of TMAO precursors and circulating TMAO levels across studies [62].

b. **Short-chain fatty acids (SCFAs)**

Acetate, butyrate, and propionate are the main metabolites produced by the fermentation of indigestible polysaccharides, and are presented as short-chain fatty acids. The beneficial effects of SCFAs are manifested not only in the gut, where they support intestinal barrier integrity by increasing the expression of cell junction proteins and serve as energy substrates for epithelial cells, but in their effect on inflammation and metabolic functions. In fact, they can affect glycemic and lipid metabolism and modulate the secretion of inflammatory cytokines and chemokines, and they exhibit anti-inflammatory and anti-tumorigenic activity [63].

Acetate is predominantly synthesized by several gut microbial taxa, particularly members of the genera *Prevotella*, *Ruminococcus*, *Bifidobacterium*, *Bacteroides*, *Clostridium*, and *Streptococcus*, as well as by *Akkermansia muciniphila* and *Blautia hydrogenotrophica*. Propionate production has been associated with microorganisms such as *Salmonella enterica* serovar Typhimurium, *Roseburia inulinivorans*, *Akkermansia muciniphila*, and *Coprococcus catus*. In contrast, the principal butyrate-producing bacteria include *Coprococcus* spp., *Faecalibacterium prausnitzii*, *Eubacterium rectale*, *Eubacterium hallii*, and *Ruminococcus bromii* [64,65,66]. Short-chain fatty acids function as important signaling molecules that interact with intestinal and extraintestinal tissues through specific cell-surface receptors. These receptors include free fatty acid receptor 3 (FFAR3/GPR41), FFAR2 (GPR43), G protein-coupled receptor 109A (GPR109A/HCAR2), and olfactory receptor 78 (Olfr78 in mice and OR51E2 in humans), as well as GPR42 and OR51E1. The binding of SCFAs to these receptors initiates intracellular signaling pathways involved in the regulation of multiple physiological processes [67]. SCFAs serve as an important energy source for intestinal epithelial cells, maintaining the integrity of the intestinal barrier, and can indirectly regulate inflammatory and immune responses, as well as blood pressure, and lipid and glucose homeostasis, through various mechanisms [10]. SCFA production, mainly butyrate, is the most abundantly reported, and plays a protective role in various conditions such as CVD, obesity, inflammation, atherosclerosis, and cancer [68]. Butyrate and propionate play a significant role in glucose homeostasis. These SCFAs have been shown to promote the secretion of intestinal hormones, including glucagon-like peptide-1 (GLP-1) and peptide YY, which enhance satiety through the gut–brain axis while also contributing to glycemic regulation by stimulating insulin release from the pancreas and suppressing glucagon secretion [69,70].

Butyrate-producing bacteria exert a protective role against coronary artery disease (CAD) risk factors such as atherosclerosis and hypertension [71]. The beneficial role of butyrate in atherosclerosis is manifested through the ability to reduce atherosclerosis formation in the aorta, reduce plaque inflammation, decrease pro-inflammatory cytokine secretion such as IL-1β and IL-10, via reduction of NF-κB [72], inhibit lipid deposition, promote macrophage accumulation, lower gut permeability [73], decrease cholesterol accumulation, and increase gut microbial diversity [74]. In cases of hypertension, butyrate demonstrates a hypotensive effect via the colon-vagus nerve signaling and GPR41/43 receptors [75], reduces gene expression of TNF-α and level of IL-6 [76], reduces endotoxemia [77], decreases gene expression of IL-1β in myocardial tissue, and inhibits cardiac hypertrophy through the inhibition of cyclooxygenase 2/prostaglandin F2 pathways [78]. Reduced production of SCFAs (mainly propionate and butyrate) in heart failure has been associated with impaired intestinal barrier integrity, systemic inflammation, endothelial dysfunction, and altered cardiac energy metabolism, potentially contributing to adverse myocardial remodeling and heart failure progression [79].

The cardioprotective effect of butyrate treatment is likely mediated by suppression of the sympathetic nervous system via the gut–brain circuit. Oral butyrate supplementation significantly reduced the infarct size, and reduced the expression of creatine kinase, creatine kinase myocardial isoenzyme, and lactate dehydrogenase [80].

The anti-inflammatory properties of butyrate are largely mediated through the inhibition of histone deacetylases (HDACs), which are involved in the regulation of innate immune signaling, myeloid cell differentiation, and inflammatory pathways associated with toll-like receptors (TLRs) and interferon-inducible genes. By suppressing HDAC activity, butyrate reduces the production of pro-inflammatory cytokines, including TNF-α, IL-12, and interferon-γ (IFN-γ), while simultaneously promoting IL-10 secretion by monocytes in vitro, thereby exerting immunomodulatory effects [81].

Short-chain fatty acids also participate in lipid and cholesterol metabolism. Propionate has been identified as a strong inhibitor of cholesterol biosynthesis, whereas the major SCFAs enhance hepatic cholesterol uptake from the circulation and promote its elimination, ultimately lowering plasma cholesterol concentrations. In addition, acetate and propionate suppress endogenous lipolysis, while propionate further modulates extracellular lipid metabolism by upregulating lipoprotein lipase expression, contributing to reduced plasma lipid levels. Together, these metabolic effects may help attenuate the risk factors involved in the onset and progression of atherosclerosis [10].

Dysbiosis is manifested by reduction of SCFA production, which is associated with hyperlipidemia and an increased risk of cardiovascular events. The gut microbiota modulates lipid profiles via SCFAs and other bioactive metabolites. SCFAs influence lipid synthesis in the liver by activating GPR41 and GPR43, regulating decrease triglyceride accumulation, and may support the synthesis of high-density lipoprotein cholesterol (HDL-C), offering protection against CVD [82].

c. **Coprostanol**

Increased cholesterol is a risk factor for CVD, as excess cholesterol in the blood and other organs comes from an imbalance between input and output. Coprostanol is an intestinal microbial metabolite produced by gut bacteria from dietary cholesterol, and its increased levels could therefore have a positive effect on health. Non-absorbable coprostanol represents the major end-product of microbial cholesterol biotransformation, and is considered clinically important due to its role in facilitating cholesterol elimination from the body [83]. Several intestinal bacteria, including *Eubacterium coprostanoligenes*, *Bacteroides* spp., *Lactobacillus* spp., and *Bifidobacterium bifidum*, can convert cholesterol into coprostanol [84,85]. The proposed pathway for microbial conversion of cholesterol to coprostanol in the microbiota involves the intermediates cholestenone and coprostanone. Recently, a microbial cholesterol dehydrogenase enzyme named intestinal sterol metabolism A (ismA) was discovered, which is encoded by the ismA genes, and is involved in the oxidation of cholesterol, via cholestenone and coprostanone, to coprostanol. The presence of ismA genes in a microbiome (bacterial group like *Oscillibacter*) is associated with the presence of coprostanol in the stool and lower levels of fecal cholesterol, offering a probable mechanism by which these bacteria may decrease host serum cholesterol levels. It highlights the potential role of cholesterol-metabolizing gut bacteria in modulating human health. Moreover, the detection of ismA genes in human metagenomes has been significantly linked to lower serum cholesterol levels, with effects comparable to those seen in genetic variants of human genes that regulate lipid homeostasis [84]. Understanding the ismA pathway could lead to probiotics or interventions that boost these cholesterol-lowering bacteria, to manage hypercholesterolemia.

By increasing the abundance of cholesterol-metabolizing gut bacteria using prebiotics, it can be possible to achieve targeted effects on host serum cholesterol, a strategy that has already been shown to be beneficial in influencing different areas of human metabolism [86]. Using coprostanoligenic strains can be of clinical interest to reduce cardiovascular risk, by modulating the gut microbiota [83]. Kazemian et al. [87] reported that *Akkermansia muciniphila* has become a key microbial player in cholesterol transformation in both healthy subjects and individuals with hypercholesterolemia and coronary artery disease. In these populations, the gut microbiota was characterized by an increased abundance of *Oscillospira*, *Ruminococcus*, *Christensenellaceae*, and *Ruminococcaceae*, along with enhanced BAs metabolism and more favorable lipid profiles, all of which are associated with a reduced risk of CVD. These findings suggest that microbial-driven cholesterol conversion may indirectly modulate cardiovascular risk, while also directly affecting intestinal inflammatory status and overall metabolic health. *A. muciniphila* and its associated metabolic pathways therefore represent promising targets for influencing cholesterol metabolism and lowering cardiovascular risk. Future approaches may aim to increase their abundance through probiotic supplementation, dietary interventions, or the administration of microbial-derived enzymes or metabolites.

d. **Phenylacetylglutamine**

Phenylacetylglutamine (PAGln) is a gut microbiota-derived metabolite that originates from the essential amino acid phenylalanine, which is found in dietary protein sources such as eggs, milk, and meat. Several members of the gut microbiota, including taxa from the families *Christensenellaceae*, *Ruminococcaceae*, and *Lachnospiraceae*, have been implicated PAGln production [88]. Dietary phenylalanine is metabolized in the large intestine by the gut microbiota to phenylpyruvatic acid. The microbial porA gene enables the conversion of phenylpyruvic acid to phenylacetic acid via two distinct metabolic routes, generating phenylacetaldehyde and phenylacetyl-CoA as intermediate products, respectively. Subsequently, phenylacetic acid is transported to the liver through the portal circulation, where it undergoes conjugation with glutamine in humans to form PAGln, while in rodents it is conjugated with glycine to produce phenylacetylglycine [89].

The level of PAGln is associated with the risk of various cardiovascular diseases and incident of major adverse cardiovascular events (MACEs) such as acute myocardial infarction, stroke, and cardiovascular mortality [90]. Recently, several studies have identified a link between PAGln and cardiovascular diseases, including coronary heart disease [91], atrial fibrillation [92], and heart failure [93]. Elevated PAGln levels are an independent risk factor for heart failure, and are associated with a higher risk of cardiovascular death. High PAGln levels are also strongly associated with a higher risk of renal dysfunction in patients with heart failure [94]. PAGln is generated in multiple organs, including the intestine, liver, and kidney, and its production depends on coordinated inter-organ communication, particularly between the gut, heart, and kidneys, which is important for maintaining metabolic homeostasis. Elevated levels of PAGln have been shown to activate adrenergic G-protein–coupled receptors (α2A, α2B, and β2), promote apoptotic processes through the TLR4–AKT–mTOR signaling axis involving toll-like receptor 4, phosphorylated protein kinase B, and mechanistic target of rapamycin, and contribute to systemic inflammation by increasing pro-inflammatory cytokines such as IL-1β, IL-6, and TNF-α. Nemet et al. [90] suggested a possible mechanism by which PAGln could promote the activation of platelets and thrombosis. PAGln activates platelet sensitivity and thrombosis potential via G-protein-coupled receptors, including α2A, α2B, and β2-adrenergic receptors.

e. **Bile acids**

Bile acids are synthesized in the liver as by-products of cholesterol metabolism. The primary BAs—cholic acid (CA) and chenodeoxycholic acid (CDCA)—are produced through the action of the enzyme cholesterol 7α-hydroxylase (CYP7A1). These primary BAs are then conjugated with the amino acids glycine or taurine, via the enzyme bile acid-CoA:amino acid N-acyltransferase (BAAT), forming conjugated BAs [95].

Bile acids are stored in the gallbladder and released into the small intestine following food intake, where they help emulsify dietary fats and improve the absorption of lipids and fat-soluble vitamins. In the terminal ileum, conjugated BAs are actively reabsorbed and returned to the liver through the portal vein, thereby completing the enterohepatic circulation. A small portion of secondary BAs is passively reabsorbed in the colon, while the remainder is excreted in the feces. Conjugated BAs enter the intestine, where they can be hydrolyzed by the microbial enzyme bile salt hydrolase (BSH), producing unconjugated BAs [96]. The gut microbiota can further transform unconjugated BAs into secondary BAs (such as deoxycholic acid (DCA) and lithocholic acid (LCA)) and tertiary BAs like ursodeoxycholic acid (UDCA). These transformations occur through reactions including 7α-dehydroxylation, dehydrogenation, and epimerization [97]. Both the gut microbiota and its metabolic products are closely associated with the severity of coronary heart disease. Certain members of the microbiota, including *Roseburia*, *Ruminococcaceae*, and *Clostridium*, play a role in regulating BAs and aromatic compound metabolism, thereby affecting the progression of coronary atherosclerosis [98].

Decrease in secondary BA excretion of deoxycholic acid and lithocholic acid is a risk factor for atherosclerotic coronary artery disease, and was found to be an independent risk factor for stroke [99].

Bile acids are signaling molecules for a range of physiological processes mediated by the nuclear farnesoid X receptor (FXR) and the membrane-bound Takeda G-protein-coupled BAs receptor (TGR5) [100,101]. FXR plays a central role in the regulation of glucose, lipid, and BA metabolism (synthesis, conjugation, and transport), including CYP7A1, and acts as a sensor of BA levels in hepatocytes and enterocytes. Activation of FXR in endothelial cells (ECs) enhances nitric oxide production and promotes vasodilation, reduces the expression of vasoconstrictive peptide endothelin-1, and modulates angiotensin II receptors, inhibiting the inflammation and migration of vascular smooth-muscle cells, a contributing factor in the progression of atherosclerotic lesions [102]. FXR expression in hepatocytes regulates FMO3, the enzyme involved in TMAO synthesis [103]. Modulating the gut microbiota by changing *Firmicutes* to *Bacteroides* allowed for a decrease in FXR activation, and dysbiosis could be strongly affecting cardiovascular health through BAs [104]. TGR5 acts as a membrane-bound BA receptor that offers protective effects against metabolic syndrome through the specific activation of intracellular signaling pathways and the maintenance of metabolic homeostasis (glucose homeostasis via GLP-1 secretion, energy metabolism, and anti-inflammatory protection). TGR5 activation in ECs and macrophages has a protective role in atherosclerosis. In ECs, the TGR5 pathway inhibits TNF-α-induced expression of vascular adhesion molecule-1 and intercellular adhesion molecule-1, and, in macrophages, inhibits the NF-κB inflammatory pathway. This mechanism reduces the production of pro-inflammatory mediators, including TNF-α, IL-1α, IL-1β, IL-6, and IL-8 [105,106]. Reduced production of secondary BAs (potentially due to dysbiosis) would lead to decreased activation of TGR5, which would accelerate the atherogenic process [107].

*The advantages and disadvantages of using metabolite concentrations in different biological samples:* disrupted bile acid homeostasis, characterized by an altered profile of circulating primary and secondary BAs, impaired signaling of FXR and TGR5, systemic inflammation, endothelial dysfunction, and myocardial remodeling, are also frequently observed in patients with heart failure [108].

The direct effects of microbial metabolites indicate that their mechanisms of action are complex, and remain incompletely understood. From a metabolic and inflammatory perspective, gut microbiota-derived metabolites, including TMAO, SCFAs and BAs, represent important mediators linking diet, host physiology, and CVD risk. These compounds are increasingly recognized not only as functional effectors of atherogenesis and systemic inflammation, but also as potential biomarkers and therapeutic targets in CVD, while their interpretation is strongly dependent on the biological matrix in which they are measured [109,110].

Plasma metabolites are most used, due to their association with systemic exposure and clinical outcomes, particularly the relationship between circulating TMAO levels and cardiovascular events. However, their concentrations are influenced by host metabolism, renal function, and inflammatory status. In the case of TMAO, impaired glomerular filtration has been shown to promote its systemic accumulation independently of gut microbial activity or dietary intake, which may complicate the interpretation of TMAO as a biomarker of CVD risk [111]. Moreover, elevated plasma TMAO levels in patients with chronic kidney disease have been associated with systemic inflammation and gut microbial dysbiosis [112].

Urinary metabolites represent a non-invasive marker of short-term dietary exposure and microbial metabolism, although they are affected by hydration and renal clearance. Postprandial studies demonstrated that urinary TMAO concentrations reflect dietary intake of choline and L-carnitine more sensitively than plasma levels [113].

Fecal metabolites provide a more direct representation of gut microbial fermentation and intestinal metabolism, particularly SCFAs associated with gut barrier integrity. Since SCFAs are rapidly absorbed and metabolized prior to entering the systemic circulation, fecal concentrations may not accurately correlate with plasma levels [114]. Nevertheless, associations between fecal SCFAs, blood pressure, metabolic syndrome, and intestinal barrier function support their relevance as markers of local microbial activity [115].

These methodological differences suggest that integrating plasma, urinary, and fecal metabolomics may provide a more comprehensive understanding of the gut–heart axis and improve the identification of clinically relevant biomarkers and therapeutic targets in CVD.

### 3.2. Mechanisms Linking Gut Microbiota to Cardiovascular Diseases—Indirect Influence Through the Immune System

The gut microbiota and the host immune system establish a dynamic, reciprocal interaction modulated by diverse molecular-signaling pathways. These include microbe-associated molecular patterns (MAMPs), bacterial metabolites, and host pattern recognition receptors (PRRs). Immune health and metabolic functions are closely linked via the gut microbiota and diet. Immune cells are constantly exposed to a wide range of microbes and compounds of microbial origin, which have important mucosal and systemic consequences [116,117]. The gut microbiota has a substantial impact on systemic inflammation, which constitutes a major contributing factor to CVD. The immunomodulatory function of the gut microbiome is presented on biological markers such as white blood-cell counts, inflammatory markers, and levels of inflammatory and anti-inflammatory cytokines [32,118].

Disruption of intestinal microbiota composition by various factors (diet and drugs) is commonly referred to as dysbiosis, and contributes to the progression of CVD by promoting major CVD risk factors, atherosclerosis and hypertension [119]. Dysbiosis is accompanied by reduced microbial diversity and changes in the relative proportions of dominant bacterial phyla. There is an increased abundance of proinflammatory bacteria, such as *Enterobacteriaceae*, *Desulfovibrio* spp., *Collinsella* spp., *Eggerthella* spp., and *Streptococcus* spp., along with a depletion of beneficial bacteria such as *Faecalibacterium prausnitzii*, *Roseburia* spp., *Akkermansia muciniphila*, *Blautia*, and *Lactobacillus*. Shifts in the *Bacillota/Bacteroidota* ratio have been linked to metabolic disturbances, and may modulate cardiovascular risk by influencing lipid metabolism, gut barrier integrity, and immune activation [120].

An important outcome of the disruption of intestinal microbiota composition is the degradation of tight junction proteins, such as zonulin and occludin, which leads to increased intestinal permeability, commonly referred to as “leaky gut.” This phenomenon permits endotoxins, such as LPS, to cross into the bloodstream and contribute to endotoxemia [28]. LPS binds to TLR4 on immune cells, activating myeloid differentiation primary response 88 and the NF-κB pathway, which plays a crucial role in regulating vascular inflammation. This activation triggers pro-inflammatory intracellular signaling pathways, leading to increased production of cytokines such as IL-1, IL-6, IL-27, and TNF-α, which contribute to systemic inflammation and endothelial dysfunction, key precursors of atherosclerosis and CVD [121,122,123].

Elevated LPS levels further stimulate the production of pro-inflammatory cytokines, including IL-6, TNF-α, IFN-γ, and IL-1β, as well as the inflammatory marker C-reactive protein (CRP). These factors promote endothelial damage, enhance monocyte recruitment to vascular sites, and contribute to chronic inflammation and vascular injury [124,125]. Circulating LPS, a component of Gram-negative bacteria from the gut, has been identified as an independent predictor of major adverse cardiovascular events (MACEs) in patients with atrial fibrillation. Reducing LPS levels, potentially through dietary interventions, represents an important therapeutic target in CVD [126]. Gut dysbiosis is linked with increased Gram-negative bacteria (*Bacteroidetes*, *Proteobacteria*, *Fusobacteria* and *Verrucomicrobia*) and increased LPS levels, which subsequently correlated with increased inflammatory markers (CRP, IL-6, TNF-α) in patients with type 2 diabetes mellitus (T2DM) and chronic kidney disease, important risk factors for the development of CVD [127]. Gut dysbiosis may promote the differentiation of T helper type 17 (Th17) cells, which secrete the pro-inflammatory cytokine IL-17, thereby contributing to vascular inflammation and plaque instability [128]. An increased number of Th17 cells in atherosclerosis promotes activation of the NF-κB pathway and stimulates the production of matrix metalloproteinases, thereby contributing to extracellular matrix degradation and increasing the risk of thrombosis and plaque rupture [129]. The correlation between gut microbiota and inflammation also occurs through platelet hyperactivation mediated by TMAO.

Activated platelets release CD40 ligand and other mediators, triggering inflammatory responses in the endothelium and resulting in endothelial dysfunction [130].

The gut microbiota immunomodulatory effects are not only limited to the modulation of cytokine secretion, but can also affect the types of cells that develop in the immune system. The lack of Th17-inducing gut bacteria led to an expansion of regulatory T cells (Tregs), which exert a crucial anti-inflammatory effect. Tregs cells have an important role in the mediation of tissue repair following cardiac injury, from the acute to chronic phase. Tregs can terminate the pro-inflammatory phase and initiate the anti-inflammatory or regenerative phase by secreting anti-inflammatory cytokines IL-10 and IL-13, and transforming growth factor-β (TGF-β) [131]. SCFAs regulate immune responses by influencing Tregs cells and reducing pro-inflammatory cytokines (IL-17, TNF-α), thus mitigating the negative effects of hypertension [132].

## 4. Gut Microbiota-Mediated Targeted Therapeutic Interventions for Cardiovascular Metabolic Diseases

### 4.1. Dietary Intervention

A growing body of evidence highlights the importance of the gut–heart axis, suggesting that modulation of the gut microbiota and support of beneficial bacterial strains may have positive effects on the cardiovascular system [133].

Dietary interventions, such as the Mediterranean diet (MedDiet), the Dietary Approaches to Stop Hypertension (DASH) diet, and a flexitarian diet, as well as the use of probiotics, prebiotics, and synbiotics, represent promising strategies for improving overall health, and may be highly effective in the prevention and management of cardiovascular disease [134].

The health benefits of the MedDiet include increased life expectancy and a reduced risk of metabolic syndrome and CVD. It also leads to notable changes in the gut microbiota, promoting the growth of SCFA-producing species—especially butyrate producers such as *Clostridium leptum* and *Eubacterium rectale*—as well as increased levels of *Bifidobacterium*, *Bacteroides*, and *Faecalibacterium prausnitzii*, while reducing the abundance of *Firmicutes* and *Blautia* species. These shifts in the gut microbiota are linked to decreases in inflammation, oxidative stress, and cancer risk, while improving overall metabolic health [135]. A one-year lifestyle intervention combining an energy-reduced MedDiet and physical activity in a Mediterranean population of older adults with overweight/obesity and metabolic syndrome improved cardiovascular risk factors, likely by altering the fecal microbiota (increased alpha diversity and reduced abundance of the genera *Eubacterium hallii* group and *Dorea*) and the metabolome, and resulted in weight loss. Key metabolites affected included BAs, ceramides, sphingosines, fatty acids, carnitines, nucleotides, and compounds related to purine metabolism and the Krebs cycle [136]. Diets rich in polyphenols and fiber, such as the MedDiet, can counteract the pro-inflammatory state associated with high-meat, low-fiber diets. A diet high in animal protein (especially red meat, eggs) may simultaneously increase the intake of TMAO precursors and reduce dietary fiber intake, resulting in a “double blow.” Low fiber intake limits the growth of beneficial bacteria, leading to reduced production of SCFAs and a weakened intestinal barrier [137,138]. To mitigate this “double blow,” dietary interventions must address both aspects of the mechanism: replacing animal protein with plant-based alternatives reduces the direct input for TMA production, while increasing fiber intake is essential. Although dietary fiber does not consistently reduce circulating TMAO levels across all individuals, it promotes a healthier gut microbiota composition that may shift microbial metabolism away from TMA production [139].

Another study presents new evidence demonstrating that fecal secondary bile acids (SBAs), such as taurolithocholic acid-3sulphate (TLCA-S) and glycocholenoic acid sulphate, modulated the effects of the MedDiet on body adiposity and lipid metabolism, which are predictive biomarkers of cardiometabolic risk for personalized dietary intervention. Their findings showed that the associations of fecal SBAs with BMI and serum lipids were modified by the presence or absence of individual microbial species, such as *Ruminococcus torques* and *Bifidobacterium longum,* which are involved in the hydrolysis of BAs [140].

Current research suggests that the beneficial effects of the MedDiet on the gut–heart axis are mediated, at least in part, by its bioactive components, particularly polyphenols derived from extra virgin olive oil and wine, as well as omega-3 polyunsaturated fatty acids (PUFAs). These compounds act through two principal mechanisms: (i) the selective shaping of gut microbiota composition and (ii) the remodeling of microbiota-derived metabolite profiles (notably SCFA and BAs, including their derivatives), which subsequently modulate host immune and metabolic signaling.

Hydroxytyrosol, a major phenolic component of extra virgin olive oil, exerts prebiotic-like effects by promoting beneficial genera (e.g., *Lactobacillus* and *Bifidobacterium*), limiting potentially pathogenic taxa, and enhancing SCFA production, thereby supporting intestinal barrier integrity and dampening inflammation via the gut–immune axis [141].

Resveratrol, a stilbene polyphenol, abundant in red wine, exhibits bidirectional interactions with the gut microbiome: it can enrich beneficial taxa (e.g., *Akkermansia*, *Bacteroides*) while suppressing opportunistic bacteria, and it is also microbially transformed into bioactive metabolites (e.g., 4-hydroxyphenylacetic acid), which mediate downstream metabolic effects via signaling pathways such as SIRT1. In addition, resveratrol exerts cardioprotective effects through SIRT1/AMPK-mediated improvement of endothelial function (via enhanced nitric oxide production), modulation of lipid metabolism with reduced LDL oxidation and triglyceride synthesis, suppression of NF-κB–driven inflammatory signaling and oxidative stress, attenuation of atherogenesis, and indirect gut microbiota-dependent mechanisms that may reduce the formation of pro-atherogenic metabolites such as TMAO [142,143].

Similarly, omega-3 PUFAs (EPA and DHA) are associated with increased microbial diversity, enrichment of SCFA-producing bacteria (e.g., *Lachnospiraceae*, *Roseburia*), reduced abundance of pro-inflammatory taxa such as *Enterobacteriaceae*, changes linked to improved mucosal barrier function, and attenuation of endotoxin-driven inflammation [144,145].

Importantly, these bioactives likely operate synergistically within the MedDiet matrix; combined polyphenol and omega-3 intake has been associated with greater antioxidant capacity and stronger anti-inflammatory effects than individual components, potentially underpinning shifts toward SCFA-producing microbiota and improved metabolic–inflammatory profiles observed with higher MedDiet adherence [146,147].

The Dietary Approaches to Stop Hypertension (DASH) eating pattern is a heart-healthy diet designed to reduce blood pressure (BP) and prevent chronic diseases [148]. This diet is an effective non-pharmacologic strategy for lowering BP, as it emphasizes fruits, vegetables, whole grains, and low-fat dairy products, while reducing sodium, sugar, and saturated fats. Zare et al. [149] reported that, in addition to lowering BP, the DASH diet significantly reduced total cholesterol (TC), LDL-C, and very-low-density lipoprotein cholesterol. The associations between adherence to the DASH diet and blood pressure and lipid levels, as well as the potential mediating role of gut microbiota, were investigated in a study by Zhang et al. [150]. Among the five DASH diet–related gut bacterial genera, *Bifidobacterium* partially explained these associations.

The flexitarian dietary pattern is characterized by a predominantly plant-based intake (vegetables, fruits, whole grains, legumes, nuts, and seeds) with occasional consumption of animal products [151]. Evidence suggests that adherence to a flexitarian diet is associated with improved lipid profiles, including lower levels of TC and LDL-C, as well as reduced BP [152]. Furthermore, emerging research indicates that plant-based and flexitarian diets may positively influence microbial composition, which could play a role in mediating their cardioprotective effects [153].

A study by Ranaivo et al. [154] included subjects at cardiometabolic risk (dyslipidemia and insulin resistance) who consumed standard bread or bread enriched with a dietary fiber mixture. In comparison with the control bread, consumption of multi-fiber bread led to a significant reduction in *Bacteroides vulgatus*, whereas it increased *Parabacteroides distasonis, Fusicatenibacter saccharivorans*, an unclassified Acutalibacteraceae, and *Eisenbergiella* (*p* < 0.01). Statistically significant reductions were observed in total cholesterol and low-density lipoprotein cholesterol (LDL-C) (*p* < 0.01), as well as insulin and homeostasis model assessment (HOMA) scores (*p* < 0.05). Enhancing the diversity of dietary fibers in everyday foods can modulate gut microbiota composition and function, and may represent a promising nutritional approach for improving cardiometabolic outcomes.

### 4.2. Probiotic, Prebiotic, and Synbiotic Intervention

Dixon et al. [155] systematically summarized randomized controlled trials (RCTs) published between 1990 and 2020 on the effectiveness of probiotic use in adults with cardiovascular disease in relation to comorbidities such as diabetes mellitus, dyslipidemia, metabolic syndrome, hypercholesterolemia, and hypertension, and cardiovascular disease risk factors like elevated blood pressure, increased parameters of the lipid profile, glycosylated hemoglobin A1c (HbA1c), fasting blood glucose (FBG), and obesity.

Meta-analysis results showed a modest effect of probiotics on systolic and diastolic blood pressure in adults with hypertension with/without type 2 diabetes mellitus [156]. The gut microbiota affects hypertension through many pathways. Hypertensive patients were shown to have lower gut microbiota alpha diversity, lower abundance of SCFA-producing microbiota, and higher abundance of Gram-negative bacteria, which are a source of pro-inflammatory LPS. Ghaffari and Roshanravan [157] reviewed the recent clinical experiments that have evaluated the biological and pharmacological activities of several types of nutraceuticals and prebiotics in preventing and treating hypertension. The benefits of dietary intervention with probiotics and prebiotics in people with hypertension are summarized by Ivanuša et al. [158].

In men with stable coronary artery disease, *L. plantarum* 299v supplementation improves vascular endothelial function and parameters of the lipid profile, and reduces inflammatory biomarkers (Table 1). Probiotics can influence genes involved in intestinal cholesterol transport and hepatic cholesterol regulation, such as those encoding 3-hydroxy-3-methylglutaryl-coenzyme A reductase [159]. A daily intake of *L. reuteri* V3041 lowered IL-6, IL-8, TNF-α, and soluble ICAM-1 levels in obese adults with metabolic syndrome, and reduced the risk of CVD [160].

Altering the composition of the gut microbiota by administering probiotics may improve metabolic dysfunction and attenuate cardiac remodeling in patients with myocardial infarction. The study results provide evidence for the efficacy and safety of manipulating the gut microbiota with probiotics *L. rhamnosus* GG in preventing cardiac remodeling after myocardial infarction. Probiotics improved metabolic endotoxemia (a reduction in LPS), inflammatory status (decrease IL-1β), and the lipid profile in patients who achieved weigh loss greater than 2.5 kg [161]. *Bifidobacterium lactis* (Probio-M8) administered to patients with coronary artery disease modulated the gut microbiota via the increase in *Bifidobacterium adolescentis*, *Bifidobacterium animalis*, *Bifidobacterium bifidum*, and *Butyricicoccus porcorum* and a reduction in *Flavonifractor plautii* and *Parabacteroides johnsonii*. Probiotics alleviated depression and anxiety in patients and reduced levels of IL-6, LDL-C and TMAO/TMA [162].

Simultaneous administration of a probiotic (*L. rhamnosus* GG, 1.9 × 10^9^ cfu) and prebiotic inulin (15 mg/day) for 60 days to patients aged 35–55 years with CAD demonstrated improvement in the composition of the gut microbiota (a significant decrease in *Firmicutes*/*Bacteroidetes* ratio) and inflammatory markers (a reduction in IL-6 and TLR-4, LPS), a decrease in malondialdehyde, and an increase in total antioxidant capacity. Probiotics and prebiotics had a more favorable effect than administered supplements alone [163].

Diabetes mellitus is a disease primarily characterized by impaired insulin secretion and action, while also triggering and sustaining metabolic changes that can lead to inflammatory processes, dyslipidemia, and hypertension, ultimately increasing the risk of serious cardiovascular complications. The effect of the multi-strain probiotic supplement LactoLevure^®^ in individuals with T2DM was evaluated in a study by Zikou et al. [164]. Adults with average age of 65 years and T2DM received one probiotic capsule of LactoLevure^®^ daily, containing *L. acidophilus* (1.75 × 10^9^ cfu), *L. plantarum* (0.5 × 10^9^ cfu), *Bifidobacterium lactis* (1.75 × 10^9^ cfu), and *Saccharomyces boulardii* (1.5 × 10^9^ cfu), for 6 months. At the end of probiotic treatment, HbA1c levels in the probiotic group (−0.73%) and the placebo group (−0.14%) were significantly reduced (*p* < 0.001). FBG decreased significantly (*p* < 0.001) in both the probiotic group (−1.39 mmol/L), and the placebo group (−0.28 mmol/L). Total cholesterol was significantly reduced (*p* < 0.01) in the probiotic group (−0.28 mmol/L) and in the placebo group (−0.01 mmol/L). Triglycerides, HDL-C and LDL-C levels were not significantly reduced. In the probiotic group, there was significantly reduced adiposity, as measured as waist circumference, versus that of the placebo group (−3.63 cm vs. −0.44 cm). The administered probiotic capsules affected several genera, metabolites, and enzymes associated with diabetes.

An individual clinical study included adult participants aged 18 years and older with T2DM, who received 25 mL of probiotic beverages containing more than 10^8^ cfu of *Lactobacillus* for 16 weeks. After 16 weeks of follow-up, participants demonstrated reductions in HbA1c and FBG levels [165]. A review and meta-analysis of RCTs, focusing on the effect of probiotics or synbiotic consumption in patients with T2DM, was presented by Nasseri et al. [166]. They showed that they could be a useful intervention for improving cardiometabolic outcomes through primary outcomes that included changes in HbA1c, and secondary outcomes that included changes in anthropometric parameters and biochemical parameters, and a reduction in inflammation and oxidative stress in patients with prediabetes and T2DM. Other studies showed that dietary supplements (probiotic and synbiotic) significantly reduced inflammatory cytokines (CRP, IL-6, TNF-α), and reduced FBG, fasting insulin, HbA1c, and homeostatic model assessment for insulin resistance (HOMA-IR), leading to improvements in glycemic markers in individuals with prediabetes and T2DM, suggesting a promising role for gut microbiota modulation in diabetes treatment [167,168].

Hypercholesterolemia is an important metabolic disorder, as well as risk factor for CVD. Participants aged 30–65 years with mild-to-moderate hypercholesterolemia (LDL-C, 100–159 mg/dL, BMI 19–30 kg/m^2^) received *Lactocaseibacillus* (*Lactobacillus*) *paracasei* susp. *paracasei* TISTR 2593, encapsulated in maltodextrin (prebiotic) in dose 1.05 × 10^9^ cfu for 90 days. After 90 days of supplementation, a significant decrease in microbial diversity was observed in the treatment group compared to the placebo group (*p* = 0.007), with the exception of increased *Flavonifractor*, suggesting a selective modulation of the gut microbiome by the probiotic. LDL-C levels were significantly lower (*p* = 0.027) in the probiotic group, while other lipid measures did not differ statistically between groups [169].

To ensure the intake of viable probiotic strains targeted at cardiovascular and other diseases through dietary supplements or food products, several factors must be considered. During their passage through the gastrointestinal tract, probiotic bacteria are exposed to unfavorable conditions, particularly in the stomach, where they encounter extremely low pH and the action of digestive enzymes such as pepsin and gastric lipase, as well as in the small intestine, where they are exposed to amylase, lipase, proteases, and bile salts [170]. An advantage of probiotic dietary supplements containing one or more bacterial strains is their precisely defined dosage. Although these products are often encapsulated in special acid-resistant capsules and technologically stabilized, it is essential to follow the recommended storage conditions, particularly temperature requirements, as well as the expiration date [171].

The consumption of probiotics, together with an appropriate food matrix, can significantly increase their survival and promote intestinal colonization. A study by Wang et al. [172] evaluated the effect of different food matrices on the survival of *Lactobacillus rhamnosus* GG during simulated digestion. The findings showed that solid carbohydrate-rich food matrices, such as durum wheat pasta, can enhance the viability of probiotics under the challenging conditions of the gastrointestinal tract. In contrast, liquid matrices such as soy milk provided less protection, due to their lower buffering capacity. The authors also emphasized the importance of the timing of probiotic administration.

Transforming clinical insights into microbiota-targeted interventions into food science requires a multi-disciplinary approach that bridges microbial physiology and food process engineering. Industrialization of these findings involves enhancing strain viability during processing, optimizing delivery systems, and designing precise dietary matrices that reduce detrimental metabolites like TMAO [173].

The industrial production of probiotic functional foods must also address the technological challenges associated with bacterial stability. The maintenance of probiotic viability in food products is mainly influenced by three groups of factors: water transformation during lyophilization, processing conditions, and physiological factors related to digestion [174,175]. The development of modern probiotic and prebiotic formulations requires a transition from single-strain products to multi-species synbiotic formulations with enhanced efficacy and stability [176].

Next-Generation Probiotics (NGPs) are targeting strains such as *A. muciniphila*, *F. prausnitzii*, or *B. fragilis*, which have shown potential to treat dysbiosis-related metabolic diseases. It is important is incorporate probiotics into fruit/vegetable juices, fermented meat, and functional snacks.

In the future it will be important to emphasize functional products based on the Mediterranean diet, and low-choline/carnitine, high-fiber, and plant-based Mediterranean diets (high in olive oil, polyphenols), which reduce TMAO levels, as well as polyphenol-rich functional foods—beverages or supplements containing plant-based compounds like apigenin or fermented-food extracts that inhibit the gut bacteria responsible for TMA production [177].

Functional “cleaner” foods involve the design of meat alternatives or modified dairy products that reduce the intake of choline and carnitine, which are the primary precursors of TMAO found in red meat and egg yolks.

Modern food technology utilizes microencapsulation systems based on alginate, chitosan, resitant starch, whey proteins, or lipid carriers, to protect probiotic strains and improve their survival in the gastrointestinal tract. Encapsulation technologies additionally allow controlled release within the intestine, and improve shelf stability in commercial products [178,179].

**Table 1:** **Pro/Pre/Synbiotics in clinical trials included in the** **review.**

Pro/Pre/Synbiotic	Dose	Subjects	Results	Ref.
*L. plantarum* 299v	20 billion cfu/6 weeks	Patients with stable coronary artery disease	Reduced inflammatory biomarkers IL-8, IL-12, and leptin. Increased plasma propionate and decreased acetate level. No effect on TMAO level.	[159]
*L. reuteri* V3401	5 × 10^9^ cfu/12 weeks	Adult patients with newly diagnosed metabolic syndrome	Decreased IL-6, IL-8, TNF-α, and s ICAM-1, increased Verrucomicrobia phylum and Akkermansia genus by *L. reuteri* V3401 treatment.	[160]
*L. rhamnosus GG*	1.6 × 10^9^ cfu/12 weeks	Patients with MI after percutaneous coronary intervention	Probiotic improved metabolic endotoxemia (reduction in LPS), inflammatory status (decrease of IL-1β) and lipid profile in patients who achieved weight loss greater than 2.5 kg.	[161]
*B. lactis* (Probio-M8)	3 × 10^10^/6 months	Patients with coronary artery disease	Probiotics alleviated depression and anxiety in patients, reduced levels of IL-6 and LDL-C, increased counts of *B. adolescentis*, *B. animalis*, *B. bifidum*, and *Butyricoccus porcorum*. Decreased counts of *Flavonifractor plautii* and *Parabacteroides johnsonii*. Serum level of TMAO/TMA was decreased.	[162]
*L.rhamnosus* GG + inulin	1.9 × 10 ^9^ cfu + 15 mg per day/60 days	Patients with coronary artery disease	Probiotic + prebiotic improved gut microbiota composition (decrease in the F/B ratio), inflammation markers (decreased IL-6, TLR-4, LPS), decreasing malondialdehyde and increasing total antioxidant capacity.	[163]
LactoLevure^®^ capsules	*L. acidophilus* (1.75 × 10^9^ cfu), *L. plantarum* (0.5 × 10^9^ cfu), *Bifidobacterium lactis* (1.75 × 10^9^ cfu), *Saccharomyces boulardii* (1.5 × 10^9^ cfu)/6 months	Patients with T2DM as risk factor of CVDs	Probiotic capsules significantly reduced levels of HbA1c, FBG (*p* < 0.001), and total cholesterol (*p* < 0.01). In the probiotic group there was significantly reduced adiposity (*p* < 0.001), As measured as waist circumference (−3.63 vs. −0.44). Triglycerides, HDL-C and LDL-C were reduced non-significantly. Probiotics affected several genera, metabolites and enzymes associated with T2DM.	[164]
*Lactobacillus* in beverages	10^8^ cfu/16 weeks	Patients with T2DM as risk factor of CVDs	Probiotic reduced levels of HbA1c and FBG.	[165]
*Lactocaseibacillus* (*Lactobacillus*) *paracasei* susp. *paracasei* TISTR 2593 encapsulated in maltodextrin (prebiotic)	1.05 × 10^9^ cfu/90 days	Participants with mild-to-moderate hypercholesterolemia	A significant decrease in microbial diversity was observed in the treatment group compared to the placebo group (*p* = 0.007), with the exception being an increased *Flavonifractor*. LDL-C levels were significantly (*p* = 0.027) reduced.	[169]

Abbreviations: LPS, lipopolysaccharides; IL, interleukin; HbA1c, glycosylated hemoglobin; TLR, toll-like receptor; TMAO, trimethylamine N oxide; HDL-C, high-density lipoprotein cholesterol; LDL-C, low-density lipoprotein cholesterol; FBG, fasting blood glucose; s ICAM-1, soluble intercellular adhesion molecule-1; T2DM, type 2 diabetes mellitus; F/B ratio, Firmicutes/Bacteroidetes; *L. Lactobacillus*; *B. Bifidobacterium*.

### 4.3. Fecal Microbiota Transplantation

Fecal microbiota transplantation (FMT) is a highly effective therapeutic strategy for modulating the composition of the gut microbiota in pathological conditions associated with dysbiosis [180,181].

FMT was originally developed to treat recurrent *Clostridium difficile* infections, and has shown promising effect in cases of extraintestinal diseases, including CVD, with existing evidence suggesting significant potential for improving cardiovascular health [182,183]. Pakmehr et al. [184] found no clinically significant changes, but many authors report more positive than negative results. Current evidence for FMT in atherosclerosis is limited, but developing. FMT from healthy WT (wild type) mice as donors to CTRP9-KO mice (the atherosclerosis-prone mouse model) can reduce plaque development, modulate systemic inflammation, and reduce TMAO, which protects against atherosclerosis progression. Conversely, the transplantation of CTRP9-KO microbiota into WT mice promoted the progression of atherosclerosis [185,186]. Larger studies focusing on atherosclerotic outcomes are needed, to clarify the therapeutic potential of FMT.

The clinical study first explores the effect of using oral FMT on hypertension with cardiovascular event-associated indicators. The study includes profiling of stool microbial composition and function, blood pressure, blood glucose and lipid levels, blood metabolite composition, use of the ankle–brachial blood pressure index and the body mass index, and assessment of adverse events, as a measure of safety [187].

FMT restores gut microbial diversity and functionality by introducing a balanced microbiota into a dysbiotic intestinal environment. It helps rebalance the *Firmicutes*/*Bacteroidetes* ratio, promotes the growth of species associated with anti-inflammatory and cardioprotective effects, and decreases pro-inflammatory cytokines. This gut microbiota modulation can increase the production of beneficial metabolites such as SCFAs, and BAs, via FXR and TGR receptors, and reduce harmful TMAO, which is involved in atherosclerosis and systemic inflammation [183,188]. FMT has shown significant impact on metabolic disorders and their CVD risk factors.

In patients with type 2 diabetes mellitus, FMT improved body mass index, HOMA-IR, and gut microbiota composition. Among these, the potential bacteria were *Chlorobium phaeovibrioides*, *Bifidibacterium adolescentis* and *Synechococcus* sp. WH8103, due to their significantly negative correlations with HOMA-IR [189]. After 12 weeks, FMT in patients with T2DM significantly reduced HbA1c% (*p* < 0.01), blood glucose (*p* < 0.01), and uric acid levels (*p* < 0.01) while increasing postprandial C-peptide levels (*p* < 0.01), despite individual variability. Patients responding to FMT have significantly higher levels of the *Rikenellaceae* and *Ruminococcaceae* families in the stool, which may serve as potential biomarkers in selecting patients with T2DM for FMT treatment [190].

Although the direct effect of FMT on cardiovascular events is still under investigation, these findings suggest that modifying intestinal dysbiosis may attenuate systemic inflammation and the metabolic imbalances that predispose individuals to CVD.

## 5. Conclusions

The interplay between the gut microbiome and cardiovascular disease is a rapidly evolving field, with significant clinical implications. This article highlights the role of gut dysbiosis in the development of systemic inflammation and metabolic dysfunction. Therapeutic strategies such as FMT, diet, and supplementation with probiotics, prebiotics, and synbiotics, show a promising effect for reshaping microbial communities and reducing the risk of cardiovascular disease. Current gaps in understanding the gut–heart axis primarily center on distinguishing causation from correlation, translating findings from animal models to humans, and establishing standardized therapeutic applications. While evidence strongly links gut dysbiosis to CVD, the precise molecular mechanisms behind these interactions remain partially unknown. It remains unclear whether gut imbalance is the cause of heart failure or ischemia and whether reduced cardiac output is a consequence of intestinal imbalance. There is a lack of long-term human studies to confirm these mechanisms.

Translating these interventions into clinical benefits remains challenging. Future efforts must focus on elucidating causal mechanisms, standardizing protocols, advancing personalized microbiota-targeted therapies, and integrating microbiome profiling into cardiovascular risk assessment. A better understanding of the gut–heart axis could open new possibilities for microbiome-based therapies that complement conventional approaches and, ultimately, improve cardiovascular outcomes and patient care.

## Data Availability

The data contained in the article are derived from public domain resources.
